# Focus on: Structural and Functional Brain Abnormalities in Fetal Alcohol Spectrum Disorders

**Published:** 2011

**Authors:** S. Christopher Nuñez, Florence Roussotte, Elizabeth R. Sowell

**Keywords:** Fetal alcohol spectrum disorders, fetal alcohol syndrome, prenatal alcohol exposure, brain, brain function, brain structure, brain abnormalities, brain imaging studies, cognitive functioning, cognitive impairment, psychosocial functioning

## Abstract

Children exposed to alcohol prenatally can experience significant deficits in cognitive and psychosocial functioning as well as alterations in brain structure and function related to alcohol’s teratogenic effects. These impairments are present both in children with fetal alcohol syndrome (FAS) and in children with heavy in utero alcohol exposure who do not have facial dysmorphology required for the FAS diagnosis. Neuropsychological and behavioral studies have revealed deficits in most cognitive domains measured, including overall intellectual functioning, attention/working memory, executive skills, speed of processing, and academic skills in children and adolescents across the range of fetal alcohol spectrum disorders (FASD). As with neuropsychological studies, brain-imaging studies have detected differences in brain structure related to alcohol exposure in multiple brain systems and abnormalities in the white matter that connects these brain regions. Several studies have found relationships between these morphological differences and cognitive function, suggesting some clinical significance to the structural brain abnormalities. Concentrations of neurotransmitter metabolites within the brains of prenatally exposed children also appear to be altered, and functional imaging studies have identified significant differences in brain activation related to working memory, learning, and inhibitory control in children and adolescents with FASD.

In the last four decades, much has been learned about the significant and permanent changes caused by the damaging effects of alcohol on the developing fetus (i.e., teratogenic effects). Fetal alcohol syndrome (FAS), for example, is the leading known and preventable cause of intellectual disabilities (Abel 1995). One recent study (Centers for Disease Control and Prevention 2009*a*) reported that 11.2 percent of pregnant women in the United States admit to any alcohol use in the previous month and 1.8 percent of these women indicated they had engaged in binge drinking during the same time frame. The estimated prevalence of FAS varies widely within the United States with cultural and ethnic differences (Abel 1995; Centers for Disease Control and Prevention 2002). Nationwide, FAS occurs in an estimated 0.5 to 7 of 1,000 births (Centers for Disease Control and Prevention 2009*a*; May et al. 2009). Prevalence varies internationally as well. For example, one recent study (May et al. 2006) estimated the prevalence of FAS to be between 3.7 and 7.4 per 1,000 births in the Lazio region of Italy, and a similar study (May et al. 2007) reported 68.0 to 89.2 per 1,000 children with FAS in the Western Cape province of South Africa.

Many alcohol-exposed children do not meet the full criteria for a diagnosis of FAS; this is usually because they are lacking the facial dysmorphology required for the diagnosis. Please refer to the article by Warren and colleagues in this issue (pp. 1–14) for a thorough review of FAS diagnostic criteria. These children have been found to have differences in brain structure (Astley et al. 2009*a*; Sowell et al. 2001*a*, *b*, 2002*a*, *b*, 2008*b*) (see figures 1, 2A, 3, and 4), and they experience significant and debilitating cognitive and behavioral impairments that impact their daily functioning (Mattson and Riley 1998; Mattson et al. 2001). The U.S. Surgeon General estimates that for every child born with FAS, three additional children are born without physical dysmorphology but still experience neurobehavioral deficits as a result of prenatal alcohol exposure (U.S. Department of Health and Human Services 2005). This estimate is likely to be revised as the technologies for assessing fetal alcohol spectrum disorders (FASD) are improved. Over the last decade, appreciation of the wide range of symptom severities has led to the development of a more inclusive term to describe these sequelae—FASD. FASD is not a diagnosis but rather an umbrella term with several more specific diagnoses. Terms used to describe individuals within the overarching category have included FAS, partial FAS (pFAS), alcohol-related neurodevelopmental disorder (ARND), fetal alcohol effects (FAE), and alcohol-related birth defects (ARBD) (Centers for Disease Control and Prevention 2009*b*). This article will refer to children with ARND, FAE, or ARBD, collectively, as having prenatal exposure to alcohol (PEA).

The effects of PEA appear to be multifactorial, and fetuses may be differentially susceptible to the same pattern, duration, timing, and dose of alcohol (Lockhart 2001). Documented accounts of twin pairs discordant for FAS have been reported in the literature, and discordance may be more prevalent in fraternal twin pairs compared with identical twin pairs (Streissguth and Dehaene 1993). These findings support either the hypothesis that genes play a role in vulnerability to the teratogenic effects of alcohol exposure in utero or that differences in placental development between twins affect the amount of alcohol a fetus is exposed to (reviewed in Warren and Li 2005). Significant variability in the range and magnitude of the effects of PEA have been reported, and factors such as dose of alcohol, exposure pattern, developmental timing of exposure, genetic variability, maternal smoking, other drug use, physical illness and infection, poor nutrition, trauma, stress, maternal age, and giving birth to children with FAS previously may all play a role in the impact of prenatal exposure on an individual child (Guerri et al. 2009). Animal studies suggest that binge drinking is associated with more severe effects than continuous consumption throughout pregnancy (Su et al. 2001) and that there is no “safe” level of prenatal alcohol exposure known in humans (Hagaman 1999; U.S. Department of Health and Human Services 2005).

This article summarizes some of the most salient neuroimaging research findings related to FASD and describes the neurobehavioral correlates of structural differences when available as well as functional brain abnormalities studied in individuals with FASD.

## Structural Neuroimaging

### Structural Magnetic Resonance Imaging and Brain–Behavior Correlations

**Figure 1:**
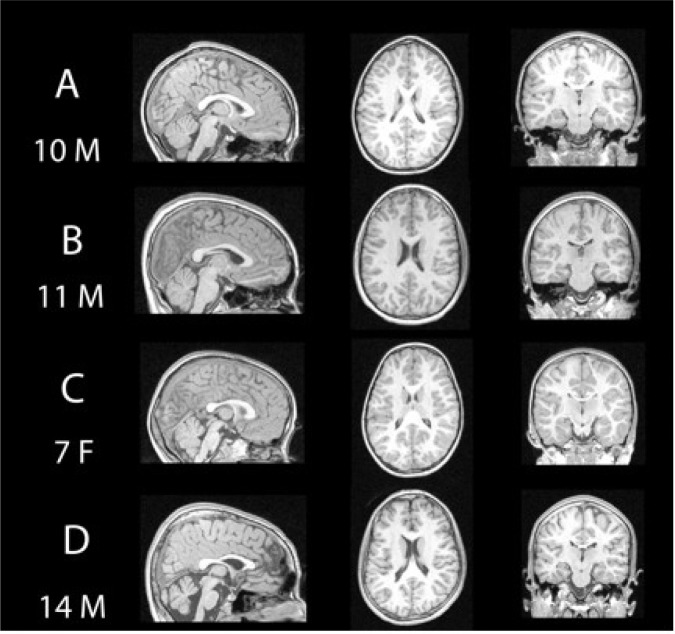
T1-weighted structural magnetic resonance imaging scans of four children demonstrating significant variability in brain structure among children with fetal alcohol spectrum disorders relative to an unexposed child. **(A)** Typically developing 10-year-old male, unexposed child. **(B)** 11-year-old male child with partial fetal alcohol spectrum disorders. **(C)** 7-year-old female child with fetal alcohol syndrome (FAS), and 14-year-old male child with (FAS) **(D)**.

Structural magnetic resonance imaging (sMRI) is a non-invasive imaging technique that allows for in vivo measurement of brain structures. Individuals are exposed to a high-intensity magnet, and, using computational techniques, researchers can capitalize on physical changes specific to different tissue types, resulting in contrast between gray and white matter within the brain (Haacke et al. 1999). Measures such as regional volume, cortical thickness, and other measures of shape can be analyzed and compared across groups. It is not currently possible to diagnose FASD based on MRI findings; all findings described below are based on group-average differences in which measures for individual subjects with FASD overlap considerably with individuals in unexposed control groups. Figure 1 shows representative structural MRI data from four individuals: one typically developing 10-year-old male, one child with pPFAS, and two children with FAS. Visual inspection of MR images do not aid in the diagnosis of FASD, even in children with complete absence of the brain structure connecting the right and left hemispheres (i.e., the corpus callosum [CC]) because this malformation is not specific to prenatal alcohol exposure (Jeret et al. 1985; Marszal et al. 2000; Shevell 2002).

#### Overall Brain Size

Postmortem studies of severely affected children with heavy prenatal alcohol exposure have consistently shown smaller head sizes as well as smaller brains (Clarren and Smith 1978; Jones and Smith 1973; Jones et al. 1973; Wisniewski et al. 1983). Neuroimaging studies have repeatedly replicated this finding in children with FAS and PEA (Archibald et al. 2001; Mattson et al. 1996; Swayze et al. 1997). Understanding localized volume reductions, however, may be more important than overall brain size in making inferences about brain systems affected most by PEA.

#### Cerebral Cortex

**Figure 2:**
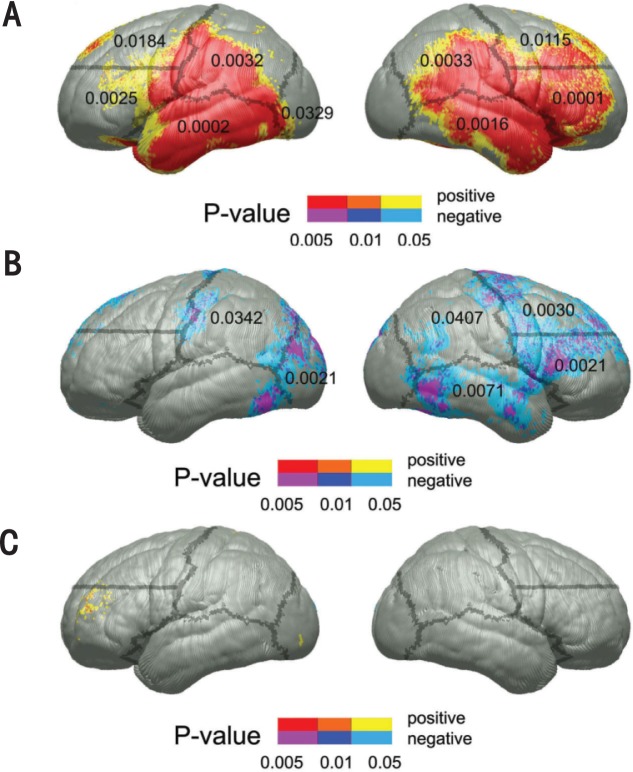
**(A)** Uncorrected P maps representing the significance of group differences in cortical thickness for the FASD versus control comparison. Positive correlations (i.e. FASD > Control) are shown in hot colors, and negative correlations are shown in cool colors. There were no regions where subjects with FASD had thinner cortex than the control subjects. Regions in red are significant at an uncorrected P value of 0.005 or less; in orange, *P* values range between 0.01 and 0.005; and in yellow, *P* values range between 0.05 and 0.01. **(B)** and **(C)** Uncorrected *P* maps representing the significance of correlations between cortical thickness and test scores of visuospatial functioning within the control group **(B)** and the FASD group **(C)**. The *P* values for positive and negative correlations are color-coded according to the color bar, with positive correlations (i.e. thinner cortex, worse performance) shown in red, orange, and yellow, and negative correlations (i.e. thinner cortex, better performance) in purple, dark blue and light blue (Sowell et al. 2007).

Researchers also have reported differences in the volume and thickness of the frontal lobes, which is consistent with neuropsychological studies that have found impairments in attention/working memory and executive functioning typically associated with frontal regions (Fuster 2003). An early study of frontal lobe volumes found them to be relatively spared when controlling for whole brain size (Archibald et al. 2001). In contrast, another study found decreased volumes of the ventral frontal lobes, most prominently in the left hemisphere, in children with FASD, with relative sparing of larger dorsal frontal regions (Sowell et al. 2002*a*). Research in children with FASD also has found increased cortical thickness in the right ventral and inferolateral frontal lobe (see figure 2) (Sowell et al. 2008*b*). Children with PEA also were shown to have increased cortical thickness in these regions, strongly suggesting that facial dysmorphology is not necessary to observe brain dysmorphology.

The parietal lobes, which generally are associated with visuospatial functioning and attention (Fuster 2003), appear to be more affected by PEA than other cortical areas. Archibald and colleagues (2001) observed gray- and white-matter volume reductions in the parietal lobes of children with FAS, and a study of children with FASD reported that the left parietal lobe had increased gray matter and decreased white matter (Sowell et al. 2001*b*). Using different methods, the same researchers found increased gray matter density in the inferior parietal/perisylvian regions bilaterally (Sowell et al. 2002*a*). A more recent study found that children with PEA had increased thickness in the parietal lobes bilaterally, suggesting that they may have “too much” gray matter in these regions when compared with unexposed children (Sowell et al. 2008*b*). White matter without a myelin sheath (i.e., insulating layer) can appear as gray matter on an MRI, suggesting excessive gray matter. Alternatively, this may be a result of incomplete pruning of neurons, which is a part of normal neural development (Sowell et al. 2004, Toga et al. 2006).

The temporal lobes usually are associated with memory formations, auditory processing, and language comprehension (Fuster 2003). Temporal-lobe volume appeared to be spared relative to other brain structures when controlling for overall brain size in children with FASD (Archibald et al. 2001), but this finding may obscure pathology within the temporal lobes that can be detected with other methods. For example, one study (Sowell et al. 2001*b*) found increased gray matter and decreased white matter in the temporal lobes of children with FASD. More recently, increased thickness in the bilateral temporal lobes also was reported, again possibly suggesting an excess of gray matter in this region (Sowell et al. 2008*b*).

As noted previously, one study reported group differences in cortical thickness between individuals affected with FASD and control subjects (see figure 2) (Sowell et al. 2008*b*). This study also examined relationships between cortical thickness and cognitive functioning. Most of the lateral brain surface in frontal, temporal, and parietal cortices showed thickness increases of up to 1.2 mm in subjects with FASD compared with control subjects. The FASD group performed more poorly than control subjects on a measure of verbal recall. Within the control group, negative correlations were found between verbal recall and cortical thickness at the superior edge of the motor strip (a band running down each side of the frontal lobe) bilaterally and in the left occipital regions. Within the FASD group, however, cortical thickness in bilateral dorsal prefrontal regions showed positive correlations with verbal recall scores. This suggests that individuals with FASD may rely more heavily on dorsal frontal structures than control subjects do, perhaps to compensate for dysfunction in other regions involved in verbal learning and recall.

On a test of visuospatial functioning, the FASD group also performed more poorly than control subjects. Within the control group, negative correlations between cortical thickness and visuospatial skills were observed in bilateral parietal, left occipital, right dorsal and ventral frontal, and temporal cortices (see figure 2B). Decreased cortical thickness associated with improved test performance might be attributed to increased cortical efficiency as unnecessary synaptic connections are pruned, and increased myelination throughout adolescence, resulting in more efficient connections between complementary brain regions. It is unclear why these negative correlations were not observed in subjects with FASD (see figure 2C). These findings suggest that pruning and myelination processes, as well as brain–behavior relationships, develop abnormally in people with FASD (Sowell et al. 2008*b*).

#### Subcortical

Subcortical structures have been associated historically with basic cognitive processes such as emotion, movement, arousal, and memory formation, but, through white-matter connections with cortical regions, they are part of higher-level networks related to more complex cognitive processes such as attention and executive functions (e.g., frontal–striatal network) (Mesulam 2000). Researchers have found decreased volumes of the basal ganglia, a group of nuclei related to motor control and learning, even when controlling for reduced overall brain size (Archibald et al. 2001; Mattson et al. 1996). The caudate nucleus, a structure within the basal ganglia associated with learning, mental flexibility, and behavioral inhibition, also was found to be significantly smaller in children with FASD (Cortese et al. 2006). The diencephalon, another region that includes several structures such as the thalamus and hypothalamus, also had reduced volumes (Mattson et al. 1996). The hippocampus is a medial temporal lobe structure essential for memory formation. One study showed relatively spared hippocampal volume, suggesting that it is either relatively spared of the teratogenic effects of prenatal alcohol exposure or that its volume is abnormally large relative to overall brain size reduction in children with FASD (Archibald et al. 2001).

#### Cerebellum

**Figure 3:**
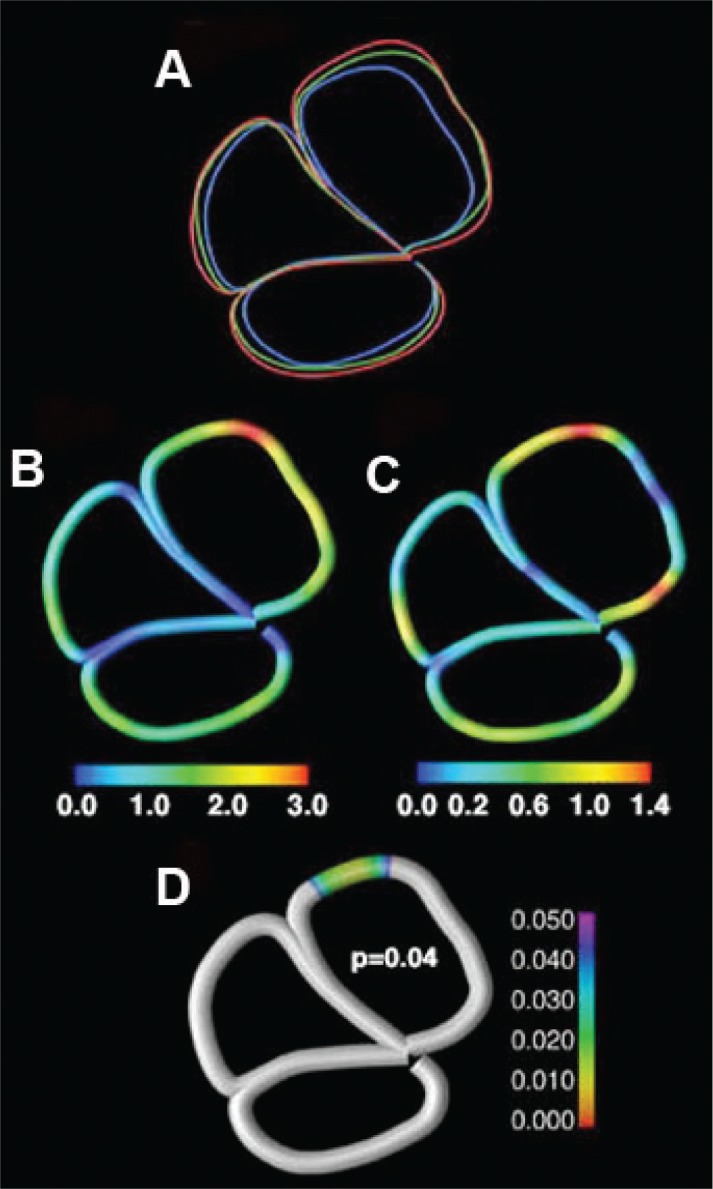
Panels **A–D** (O'Hare et al. 2005): **(A)** Individuals with prenatal exposure to alcohol (PEA) (green) and (FAS) (blue) show pronounced displacement of the anterior vermis relative to control subjects (red). **(B)** Amount of displacement among alcohol-exposed individuals compared with control subjects. **(C)** Amount of displacement among PEA individuals compared with control subjects. Color coding in panels **B** and **C** refers to the amount of displacement (in millimeters) according to the color bars below each panel. **(D)** Anterior vermal regions showing the greatest amount of displacement overlap with those regions significantly correlated with a measure of verbal learning within the alcohol-exposed group. This statistical map shows the locations of significant correlations between total learning score and displacement magnitude (greater displacement-worse performance). Note the partial overlap between the significant region in this map and the region showing greatest amount of displacement in panel **B**. The color on the map **D** represents the *p* value according to the color bar to the right of **D**. Regions in gray were not statistically significant.

The cerebellum now is known to play a role in attention, executive functions, and other complex tasks as well as movement (Strick et al. 2009). O’Hare and colleagues (2005) examined structural abnormalities in the narrow structure between the right and left hemispheres of the cerebellum (i.e., cerebellar vermis) of individuals with FASD and tried to evaluate some of the cognitive correlates of vermal dysmorphology (see figure 3). The study participants were tested on verbal learning, thought to be cerebellar dependent, and on visuospatial memory, thought to be cerebellar independent. The researchers found abnormalities in the size and location of the vermis in people with FASD. There was a statistically significant reduction in the area of the anterior vermis in alcohol-exposed subjects and a reduction in posterior–inferior lobe area. Alcohol-exposed individuals showed greatest displacement of the anterior vermis. The anterior vermis appeared to shift inferiorly and posteriorly compared with the control group. This finding is consistent not only with earlier human studies of prenatal alcohol exposure showing a significant reduction in the area of the anterior vermis, whereas posterior regions appeared relatively spared (Sowell et al. 1996), but also with animal studies reporting more Purkinje cell loss in the anterior vermis (Goodlett et al. 1990).

The superior edge of the anterior vermis showed the strongest negative correlation with verbal learning performance in the alcohol-exposed group (see figure 3D). This region partially overlapped with the vermal region showing the greatest amount of displacement in the alcohol-exposed group. This suggests that one cognitive correlate of cerebellar vermal displacement is impaired verbal learning. Correlations between vermal morphology and visuospatial memory performance were not significant, suggesting some specificity to the verbal cognition–vermal morphology relationship (O’Hare et al. 2005).

#### Corpus Callosum

In general, there is a higher incidence of midline brain anomalies in FASD compared with other brain regions (Bookstein et al. 2002*a*; Swayze et al. 1997), and midline structures, including facial features, are believed to be more vulnerable than more lateral structures to the effects of PEA. Many defects in the development of the corpus callosum (CC) have been observed, including agenesis, decreased local volumes, and other developmental anomalies (Riley et al. 1995; Swayze et al. 1997). Bookstein and colleagues (2001) reported that a high level of variability in the shape of the CC among children with FASD is a distinguishing characteristic from unexposed children, and this has allowed for a high level of classification accuracy of subjects based on callosal morphology. This high level of CC shape variability also was found in an adult sample of people with FASD (Bookstein et al. 2002*b*).

**Figure 4:**
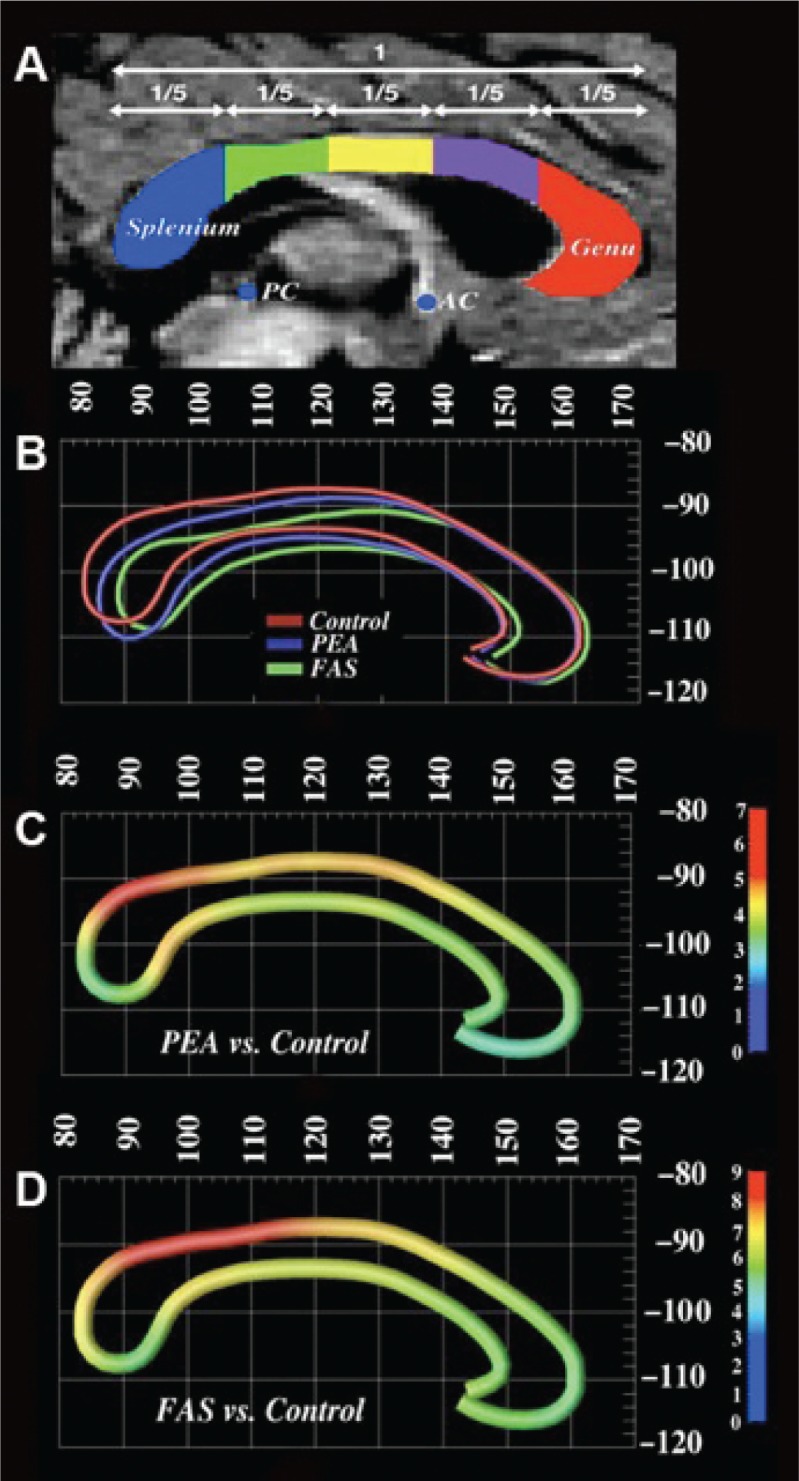
**(A)** (Sowell et al. 2001*a*): Corpus callosum divided into five equal lengths along the horizontal axis. The splenium is blue, isthmus is green, posterior midbody is yellow, anterior midbody is purple, and the genu is red superimposed over the grayscale midsagittal slice. **(B)** Average callosal lines in ICBM-305 standard space shows the distinctions between FAS, PEA, and control groups but with similar pattern of displacement. Note the placement of the corpus callosum in the PEA group somewhere between that of the control and FAS groups. **(C)** Map (in ICBM-305 space) shows average displacement vectors in millimeters between the PEA and control groups and between FAS and control groups **(D)**. Again, the pattern of displacement is the same in the PEA subjects, but the displacement is somewhat less severe than that observed for the FAS subjects.

Other researchers found structural abnormalities in the location and size of the CCs of children, adolescents, and young adults with FASD and attempted to identify cognitive correlates of callosal dysmorphology by evaluating verbal learning abilities and visuospatial skills in these subjects (see figure 4) (Sowell et al. 2001*a*). In the FASD group, the inferior and anterior displacement was mainly localized to posterior splenial and isthmus regions of the CC, whereas the more anterior genu and midbody regions were relatively spared. Individuals with FASD also showed a reduction in the area of the CC, and the most posterior region (splenium) was much more affected than any of the more anterior regions.

Subjects with FASD performed more poorly than control subjects on a test of verbal learning. The researchers reported relationships between verbal learning and the location of the CC in the FASD group (Sowell et al. 2001*a*). The more the CC was displaced in the anterior direction, the worse the verbal learning performance. Correlations between CC dysmorphology and measures of visuospatial functions were much weaker than correlations between callosal dysmorphology and verbal learning functions.

Another study (Dodge et al. 2009) of children with FASD tested the hypothesis that callosal abnormalities associated with heavy prenatal alcohol exposure impair the functioning of the CC. Researchers assessed the efficiency of interhemispheric transfer of tactile information in school-aged children with FASD from Cape Town, South Africa (Dodge et al. 2009). Children with FASD made significantly more transfer-related errors than children without an FASD diagnosis, and there was a negative correlation between the number of transfer-related errors and the size of the isthmus and the splenium in children with FASD and control children.

These results are consistent with previous studies that have documented abnormalities in callosal morphology in children with FASD. For example, Sowell and colleagues (2001*a*) found that the posterior regions of the CC were much more affected by PEA than anterior regions. This study reported correlations between difficulty in transferring tactile information across the CC and area reductions in the isthmus and splenium; no significant correlations were detected for more anterior CC regions (Dodge et al. 2009). This study thus expands on previous reports by showing that a reduction in size of these regions has a functional impact.

### MR Spectroscopy

Compared with other neuroimaging techniques, MR spectroscopy (MRS) has been relatively underutilized in studying FASD, and the few existing studies have had somewhat inconclusive results. Using MRI technology, MRS capitalizes on different physical properties of chemicals in the body when they are exposed to a high magnetic field, and in brain research, measures of brain chemicals that are important to specific processes can be detected (Ross and Bluml 2001; Ross and Sachdev 2004). For example, concentrations of choline (Cho), believed to be a marker of myelination and cell membrane stability, *N*-acetyl aspartate (NAA), a neuronal or axonal marker, and creatine (Cr), a marker of metabolic activity, can be measured either in absolute concentration values or as ratios relative to each other.

One study that examined metabolite ratios in multiple brain structures of children with FASD and control subjects found lower NAA/Cho and NAA/Cr ratios in parietal and frontal cortices, frontal white matter, the CC, thalamus, and cerebellar dentate nucleus of children with FASD (Fagerlund et al. 2006). The investigators concluded that the brain metabolism of children with FASD is altered permanently in multiple brain regions.

Other researchers (Cortese et al. 2006) detected a higher ratio of NAA/Cr in the caudate nucleus of children with FASD compared with control subjects, and this was primarily related to an increase in NAA. This increase in NAA was thought to indicate a lack of normal cell death, dendritic pruning, and/or myelination.

A more recent MRS analysis observed significantly lower concentrations of choline in frontal/parietal white matter regions lateral to the medial corpus callosum (Astley et al. 2009*b*). As frontal white matter volumes and CC length decreased, choline concentrations also decreased. Decreased choline concentrations also were associated with more severe FASD diagnoses and increased impairment in neuropsychological test performance.

### Diffusion Tensor Imaging

Diffusion Tensor Imaging (DTI) also uses MRI technology and allows measurement of the diffusion of water molecules within brain tissues, specifically white matter, in order to produce images of white matter tracts and measures of white matter integrity (Basser et al. 1994; Le Bihan 1995). Fractional anisotropy (FA) is a weighted measure of water diffusion and is believed to be an indicator of the integrity of white matter, with higher values indicating increased integrity (i.e., increased myelination, increased axonal density or diameter). Mean diffusivity (MD) is a measure of total diffusion, and higher MD suggests poorer integrity (i.e., less myelination, lower density or disorganization of axonal fibers). DTI focuses on white matter throughout the brain, and, surprisingly, studies have not found differences in diffusion properties within the CC, which has been shown to have morphological differences in structural MRI studies.

Research with young adults with FAS has found lower FA values in the genu, splenium (Ma et al. 2005), and isthmus of the CC (Li et al. 2009). In a study of children with FASD, Wozniak and colleagues (2006) measured lower MD in the isthmus of the CC, which suggests microstructural abnormalities in the posterior CC even in children without the facial dysmorphology for an FAS diagnosis. Prenatal exposure to alcohol was associated with lower FA in several brain regions, including the body of the CC and white matter tracts in bilateral medial frontal and occipital regions (Fryer et al. 2009).

Researchers using a combination of DTI and T1-weighted MRI to evaluate white matter integrity in people with FASD attempted to relate these findings to neurocognitive deficits, in particular visuomotor integration and reading skills (Sowell et al. 2008*a*). FA reflects the quality of or organization of white matter microstructures, but this particular study also evaluated group differences in white matter density in order to assess white matter macrostructure (i.e., the location and amount of white matter in the brain.) The most notable significant clusters where FA was significantly lower in the FASD group than in the control group were in regions of the lateral splenium of the CC (medial superior parietal white matter), posterior cingulate white matter bilaterally, and in the deep white matter of the right temporal lobe. White matter density appeared to be lower in the FASD group in some, but not all, regions where FA was affected.

Although correlations between reading scores and FA were not significant in any region tested, it appeared that lower FA values in the lateral splenium and parietal white matter bilaterally were associated with worse performance on a test of visuomotor integration, but only within the FASD group (Sowell et al. 2008*a*). This is consistent with the role of the lateral splenium in visuospatial processing. This finding suggests that some white matter microstructural abnormalities (reduced myelination or fiber disorganization) in individuals with FASD appear to be clinically significant. However, it is difficult to infer from human DTI studies whether myelin deposition and organization of white matter fibers is a prerequisite for intact visuomotor integration or whether experience and facility at visuomotor integration leads to higher myelin deposition and better fiber organization.

### Summary of Structural and Brain–Behavior Findings

When reviewing sMRI studies of children with FASD, it appears that many brain systems are affected by the teratogenic consequences of prenatal alcohol exposure (see figures 1, 3, and 4). Earlier findings such as volumetric reductions in overall brain size, subcortical structures, the cerebellum, and parietal lobes initially suggested that other brain regions were relatively spared. More recent studies, however, suggest that structures previously believed to be spared also are affected. Enhanced sensitivity resulting from improvements in MRI data acquisition and analysis technologies may underlie these newer findings. For example, differences in cortical thickness in temporal and frontal regions were observed with the use of novel whole brain methods that allow for greater spatial precision (Sowell et al. 2002*b*, 2008*b*), which may have been obscured in previous studies where gray matter was evaluated across the entire frontal lobe. It is conceivable that relative “sparing” measured by higher volumes when larger lobar regions are evaluated is actually a reflection of a pathological process such as incomplete cortical pruning during childhood and adolescence, which could result in apparent larger volumes in individuals with FASD. Clearly, the brain-imaging studies show that “more” is not necessarily better when it comes to cortical thickness and regional brain volumes. Alternatively, it is possible that some brain regional volume or thickness increases in children with FASD are compensatory for other brain regions functioning poorly as a result of volume decrements.

Although the focus of many sMRI studies has been on morphometric differences between people with and without FASD, a handful of studies have successfully integrated what is known about function from neuropsychological studies with brain structure. Differences in specific brain structures do not necessarily translate directly to knowledge about differences in cognitive function given the complex and interrelated anatomical systems within the brain. For example, it is possible that relationships between cognitive function and frontal lobe brain structure actually are mediated by striatal dysmorphology, and relationships between brain and behavior are thus likely indirect in some instances. Most studies have attempted to investigate brain–behavior relationships through correlational methods on a structure-by-structure basis, and although these findings are important, interpretations are limited by the nature of correlational research. Future studies may benefit from investigating connectivity (e.g., see Aron et al. 2007) throughout the brain as it correlates with cognitive function in various domains.

### Functional Brain Abnormalities in FASD

One of the most recent and most frequently used techniques to study brain function in humans is functional MRI (fMRI), a noninvasive method that allows researchers to localize functional brain activation with an accuracy of millimeters and a temporal resolution of seconds. This technique measures the dynamic regulation of blood flow in the brain (i.e., the hemodynamic response) related to neural activity. Blood releases oxygen to firing neurons at a greater rate than to inactive neurons. The MR signal of oxygenated hemoglobin is different from the MR signal of deoxygenated hemoglobin. These transient changes in MR signal, which accompany hemodynamic events, can be observed when nuclear magnetic resonance images of the brain are acquired in rapid succession. The brain regions showing an increase in the intensity of the MR signal correspond to focal areas of activation (Cohen and Bookheimer 1994). Only five known fMRI studies of FASD have been published to date, which are described below.

### Spatial Working Memory

The first fMRI study that examined brain function in adults and children affected by FASD evaluated brain activation during a spatial working memory (WM) “N-back” task^1^ (Malisza et al. 2005). Behavioral results indicated that people with FASD showed a different response pattern than control subjects, with fewer correct responses, longer latencies during correct responses, and higher rates of nonresponding, but the pattern of responding was similar for children and adults within each group.

fMRI results indicated that both children and adults with FASD showed greater activity than control subjects in the inferior and middle frontal cortex (Malisza et al. 2005). This suggests improper functioning of prefrontal areas, which are involved in executive functions. In addition, non-FASD children showed increased frontal lobe activity with increasing task difficulty, whereas this pattern was not consistently observed in children with FASD. In adults with FASD, there was a trend toward greater brain activity with increasing task difficulty but this pattern was much weaker than in the control group. These group differences in frontal lobe activation, which appeared to be similar in children and adults, are consistent with the behavioral findings suggesting few age-related improvements. Abnormal activation also was observed in the parietal cortex, where greater activation was reported in control subjects than in children with FASD in one of the task conditions. As is true with most neurodevelopmental studies in which the patient group’s task performance is impaired relative to control subjects, interpretation of results must remain cautious because both accuracy and reaction times were poorer in the FASD group than in control subjects. This makes it difficult to determine whether differences in brain activation reflect general factors related to performance or specific group effects.

### Inhibitory Control

Streissguth and colleagues (2004) recently investigated the neural bases for the disinhibited behavioral profile that frequently characterizes people with FASD and contributes to dysfunction in school, employment, and social settings. On the basis of evidence from functional neuroimaging, animal models, and human lesion studies suggesting that prefrontal cortical regions are involved in inhibitory control and that subcortical modulation of response inhibition occurs (via frontal-subcortical loops that link the basal ganglia to the frontal lobes), researchers hypothesized that people with FASD would show differences in functional activation within frontal-striatal brain regions during a response inhibition “go/no-go” task^2^ (Fryer et al. 2007).

fMRI results strongly supported this hypothesis. Despite equivalent behavioral performance, study participants with FASD showed increased functional activation relative to control subjects in the prefrontal cortex and decreased activation in the caudate nucleus during trials that required response inhibition (Fryer et al. 2007). This finding suggests that the frontal-striatal circuitry thought to mediate inhibitory control is sensitive to alcohol teratogenesis. The increased recruitment of prefrontal regions may mitigate decreased frontal-striatal network efficiency induced by alcohol teratogenesis. Because prefrontal cortical activation associated with inhibitory control becomes more focused over the course of typical development (Tamm et al. 2002), these results are suggestive of an immature pattern of prefrontal engagement in individuals with FASD. Alternatively, individuals with FASD may overactivate prefrontal cortical regions when engaging inhibitory control in order to offset the effects of structural damage to striatal regions.

### Verbal Learning

**Figure 5:**
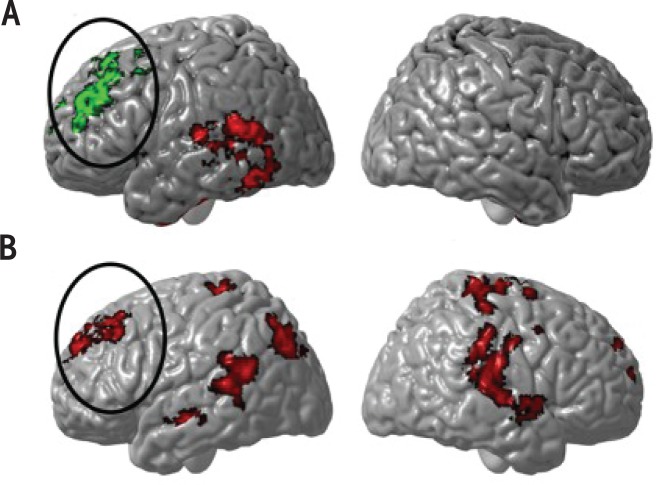
**(A)** Group differences in brain activation during verbal learning between the alcohol-exposed and the entire control group. Z values are displayed in color with voxel thresholds of Z>1.7 and a (corrected) cluster significance threshold of *P*=0.05. Significant alcohol-exposed > control regions are shown in green, and control>alcohol-exposed regions are shown in red. Note the significant increase in activation in the left frontal lobes (in green inside the black oval) in the alcohol-exposed relative to controls (Sowell et al. 2007). **(B)** Group differences in brain activation during verbal working memory between alcohol-exposed subjects and control participants when IQ differences between these groups are statistically controlled. Shown here are regions of significant (Z >1.7 and a corrected cluster significance threshold of *P*=0.05) activation for verbal WM vs. rest contrast. Regions in red represent regions where alcohol-exposed subjects display greater activation relative to typically developing subjects (O'Hare et al. 2009). Note the anatomical similarity of the increased activation in dorsal frontal lobes (green in **A** and red in **B**) for both fMRI tasks.

Given that neuropsychological studies have shown verbal learning deficits, and because brain structures involved in verbal learning show structural abnormalities in children with heavy prenatal alcohol exposure, research has examined fMRI activation patterns corresponding to verbal paired associate learning (PAL) in children with heavy prenatal alcohol exposure (see figure 5) (Sowell et al. 2007). fMRI results indicated that individuals with FASD showed significantly less activation than control subjects in the left medial and posterior temporal regions and significantly more activation in the right dorsal frontal cortex, even when group differences in IQ and memory performance were statistically controlled.

This pattern of increased frontal and decreased medial temporal activation may be consistent with the interpretation that people with FASD rely more heavily on frontal memory systems to encode and retrieve verbal information, perhaps compensating for dysfunctional medial temporal memory systems, which have been reported in previous studies to show structural abnormalities such as increased gray matter volume (Archibald et al. 2001). In addition, individuals with FASD showed functional activation abnormalities in posterior temporal regions, where increased gray matter also has been previously reported (Sowell et al. 2002*a*). Thus, as with medial temporal lobe regions, increased gray matter in the posterior perisylvian regions does not seem to be associated with sparing of function in individuals with heavy PEA.

### Verbal Working Memory

An fMRI study of verbal working memory (WM) function in children and adolescents affected by FASD (see figure 5) (O’Hare et al. 2009) also was conducted in some of the same children studied with a verbal learning fMRI paradigm described above. fMRI results indicated that both study groups engaged cerebro-cerebellar networks in response to a verbal WM challenge, but the extent to which the two groups relied on this network of brain regions differed. Despite equivalent performance between groups, individuals with FASD showed increased activation relative to control subjects during verbal WM in left dorsal frontal and left inferior parietal cortices and in posterior temporal regions bilaterally. Thus, this study provides evidence for WM-related functional activation abnormalities in posterior temporal and inferior parietal regions, two brain areas that have been shown in previous studies to have increased gray matter in individuals with FASD (Sowell et al. 2002*a*).

In addition, individuals with FASD showed increased dorsal frontal activation relative to control subjects (see figure 5B), which is consistent with the reports summarized above describing altered functioning in this region during verbal learning (Sowell et al. 2007) and response inhibition (Fryer et al. 2007). These results suggest that individuals with FASD recruit a more extensive network of brain regions during verbal WM relative to control subjects, possibly because fronto-parietal processing during verbal WM may be less efficient in these individuals. These findings are suggestive of functional activation recruitment abnormalities, both in regions known to be structurally abnormal (inferior parietal and posterior temporal) and in regions thought to be relatively structurally normal (left dorsal frontal cortex) in people affected with FASD.

### Visual Working Memory

In the most recent study to report abnormal functional brain activation patterns in individuals with FASD, investigators used fMRI to study brain activation during performance on a visual N-back WM task (Astley et al. 2009*a*). One of the goals was to determine whether fMRI could distinguish diagnostic subclasses within the FASD spectrum. Study participants were diagnosed using the FASD four-digit code (Astley and Clarren 2000). In order of decreasing severity, the four experimental groups were FAS/pFAS, static encephalopathy/alcohol-exposed (SE/AE), neurobehavioral disorders/alcohol-exposed (ND/AE), and healthy control subjects with no PEA. Behavioral results indicated that performance decreased and reaction time increased linearly with increasing severity of FASD group membership using these diagnostic categories. Children within all diagnostic subclasses showed significant WM impairments on this task.

fMRI results indicated that, when considering 2-back task activation patterns (i.e., those achieved with an N-back task with *n* = 2) the FAS/pFAS group had significantly lower activation than control subjects in various areas that are part of the neurocognitive network involved in WM, such as the right posterior parietal lobe, right dorso-lateral prefrontal cortex (DLPFC), and right middle frontal regions. Progressing across the four groups from FAS/pFAS to control subjects, activation levels increased in these brain regions. It appeared that higher activation in the DLPFC and middle frontal gyrus in both hemispheres was related to lower numbers of errors on the 2-back task. These results suggest that children across the full spectrum of FASD show significant WM deficits, which are correlated with abnormalities in activation in brain regions known to be involved in WM.

## Summary of fMRI Findings

The fMRI findings summarized above demonstrate significant differences in brain activation between FASD and control groups on various tasks that involve higher-order cognitive processes. These functional neuroimaging results are thus consistent with the neuropsychological literature, which indicates that individuals with FASD show impairments in a wide variety of cognitive domains. The fMRI studies published to date suggest that people with FASD show abnormal patterns of brain activation during tasks involving spatial WM (Malisza et al. 2005), response inhibition (Fryer et al. 2007), verbal learning (Sowell et al. 2007), verbal WM (’Hare et al. 2009), and visual WM (Astley et al. 2009*a*). Consistent across most of the studies and samples evaluated by multiple research groups, there appears to be increased activation in dorsal frontal cortices in children with FASD (see figure 5), regardless of fMRI task (Fryer et al. 2007; Malisza et al. 2005; O’Hare et al. 2009; Sowell et al. 2007). It is not clear why increased frontal activation was not reported in one fMRI study of WM (Astley et al. 2009*a*), because WM typically requires frontal cortex involvement. Perhaps regions of interest studied in that report did not capture frontal regions classically involved in WM, which may have required whole-brain analyses, more typically reported in the current literature.

To increase our confidence in fMRI results, future studies using whole-brain methodologies and direct statistical comparisons of group effects should replicate these findings with larger study populations. Additional studies also are needed to determine whether abnormal patterns of brain activation also are observed in these patients in other cognitive domains (such as attention, language comprehension and fluency, planning and problem solving, etc) and to understand how these functional brain abnormalities relate to cognitive performance and behavioral outcomes.

## Summary and Conclusions

Neuropsychological studies have demonstrated that nearly every cognitive domain evaluated is affected by prenatal exposure to alcohol and that these deficits are present in children with FAS and PEA. sMRI studies of children with FASD show that many brain systems are affected by the teratogenic effects of PEA, including structures previously believed to be relatively spared. Several studies have attempted to evaluate relationships between brain structure and cognitive functioning and found that these relationships were altered as a function of PEA. Consistent with the neuropsychological and structural MRI literature, fMRI findings demonstrate significant differences in brain activation between FASD and control groups on a variety of cognitive tasks. However, all these studies have limitations, and it is important that future investigators replicate the results presented above in larger samples of participants in order to increase power. Most studies reviewed utilized case–control methodology, which has both advantages and disadvantages. Although this methodology allows the study of relatively rare conditions such as FASD and may be the only practical method, exposure and other history are obtained by interview, which is subject to recall bias. It is important to note that in some cases validation of exposure may be incomplete or impossible to obtain. An important goal for future studies will be to continue integrating our understanding of structure and function, and it also will be essential to continue using multimodal methods that combine neuropsychological tools and structural and functional neuroimaging.

## Footnotes

1 In the N-back task the subject is presented with a sequence of stimuli and asked to indicate when the current stimulus matches the one from n steps earlier in the sequence.

2 Go/No-Go reaction time tasks require that the subject press a button when one stimulus type appears and withhold a response when another stimulus type appears.
